# Comparing research and development, launch, and scale up timelines of 18 vaccines: lessons learnt from COVID-19 and implications for other infectious diseases

**DOI:** 10.1136/bmjgh-2023-012855

**Published:** 2023-09-11

**Authors:** Wenhui Mao, Armand Zimmerman, Elina Urli Hodges, Ernesto Ortiz, Galen Dods, Andrea Taylor, Krishna Udayakumar

**Affiliations:** 1 Duke Global Health Innovation Center, Duke Global Health Institute, Durham, North Carolina, USA; 2 Innovations in Healthcare, Durham, North Carolina, USA; 3 Center for Policy Impact in Global Health, Duke Global Health Institute, Durham, North Carolina, USA; 4 Science and Society, Duke University, Durham, North Carolina, USA

**Keywords:** COVID-19, Vaccines

## Abstract

Over the next decade, millions of deaths could be prevented by increasing access to vaccines in low-income and middle-income countries (LMICs). The COVID-19 pandemic has demonstrated that the research and development (R&D), launch and scale up timelines of vaccines can be drastically shortened. This study compares such timelines for eighteen vaccines and identifies lessons and implications for accelerating the R&D, launch and scale up process for other vaccine candidates. To replicate the rapid R&D process of the COVID-19 vaccines, future vaccine R&D should capitalise on public–private knowledge sharing partnerships to promote technology innovation, establish regional clinical trial centres and data sharing networks to optimise clinical trial efficiency, and create a funding mechanism to support research into novel vaccine platforms that may prove valuable to quickly developing vaccine candidates in future global health emergencies. To accelerate the launch timeline, future efforts to bring safe and efficacious vaccines to market should include LMICs in the decision-making processes of global procurement and delivery alliances to optimise launch in these countries, strengthen the WHO prequalification and Emergency Use Listing programs to ensure LMICs have a robust and transparent regulatory system to rely on, and invest in LMIC regulatory and manufacturing capacity to ensure these countries are vaccine self-sufficient. Lastly, efforts to accelerate scale up of vaccines should include the creation of regional pooled procurement mechanisms between LMICs to increase purchasing power among these countries and an open line of clear communication with the public regarding pertinent vaccine information to combat misinformation and vaccine hesitancy.

**Summary box**

- The end-to-end scale up timeline varied substantially among different vaccines. However, the COVID-19 pandemic has demonstrated that the research and development (R&D), launch and scale up timelines of vaccines can be drastically shortened and lessons can be applied to other vaccines.
- Funding the development of novel vaccine platforms, establishing regional clinical trial centres and data sharing networks, and forming public–private knowledge sharing partnerships are all strategies that may accelerate the R&D of new and existing vaccine candidates.
- Including low-income and middle-income countries (LMICs) in the decision-making processes of global procurement and delivery alliances, strengthening the WHO prequalification and Emergency Use Listing programmes, and investing in LMIC regulatory and manufacturing capacity will likely bring LMICs closer to a state of vaccine self-sufficiency thereby reducing delays to launching new and existing vaccines in these countries.
- Creating regional pooled procurement mechanisms between LMICs, including LMICs in global supply chain decision-making, and keeping an open line of clear communication with the public regarding pertinent vaccine information may increase uptake of new and existing vaccines in LMICs and other countries worldwide.

## Introduction

The COVID-19 pandemic revealed that vaccine development, launch and scale up timelines can be shortened drastically. Some studies have identified key factors that facilitated the early development of COVID-19 vaccines.^1–3^ However, even after being successfully developed, there are multiple hurdles ahead for vaccines to reach target populations. The world is at the end of the emergency response stage of the COVID-19 pandemic, but there are other vaccine-preventable diseases (VPDs) that need further attention. The goal of this study was to compare the research and development (R&D), launch and scale up timelines of COVID-19 and non-COVID-19 vaccines and derive key lessons learnt from the COVID-19 pandemic and their implications for the development, launch and scale up of vaccines for other diseases.

## Why it is important to accelerate the development and scale up of vaccines?

Vaccination remains one of the most cost-effective public health interventions available to us today.^4 5^ The benefits of vaccines reach beyond disease control and include cost savings from averted health expenditure, increased productivity, increased educational attainment, reductions in socioeconomic and gender inequalities, and increased economic growth.^6 7^ Despite the availability of vaccines for over 25 infectious diseases, these diseases remain a substantial burden to low-income and middle-income countries (LMICs).^8^ In 2019, 14.9% of global deaths were caused by infectious diseases and 93.4% of all deaths from infectious diseases occurred in LMICs.^9^ Moreover, in 2018, 700 000 children under the age of 5 years died from vaccine preventable infectious diseases, 99.0% of whom lived in LMICs.^10^ Further action is needed to scale up vaccines in LMICs and ensure preventable morbidity and mortality are not incurred.

Current efforts to develop and scale up vaccines to countries with the greatest need are slow. Recent projections show that only 5 of 195 countries measured are on track to attain Sustainable Development Goal 3 which calls for 100% coverage of seven common vaccines (diphtheria–tetanus–pertussis-3, measles, polio, hepatitis B, Haemophilus influenzae type b, pneumococcal conjugate and rotavirus) among all at-risk populations by 2030.^11^ Progress is also slow for new vaccines in development that the total time between antigen identification and market entry of a new vaccine can take 10–15 years.^12^ Failure to accelerate the expansion of access to both existing and new vaccines could result in millions of preventable deaths. Targeted investments, for example, to develop and scale up new and existing technologies that address infectious diseases could avert 10 million deaths by 2035 with a high return on investment.^13^ Evidence showed that countries with poorer logistics performance generally have more challenges scaling up vaccines.^14^ Without extra effort to improve ‘last-mile’ vaccine delivery, poorer countries would lag behind. In addition to protecting individuals from specific diseases, vaccines can also protect broader communities through herd immunity. While the vaccination rate needed for herd immunity varies by disease and vaccine, it is normally a relatively high level such as 80% to obtain herd immunity against polio, for example.^15^

There is no standardised end-to-end scale up pathway for vaccines and many factors could affect the development, launch and scale of vaccines. To start with, VPDs are more prevalent in LMICs and there is a lack of economic incentives for developers to invest in R&D for those vaccines.^16 17^ Vaccine manufacturers also face challenging introduction, or launch, processes including ambiguous regulatory policies and procedures in different countries, which could lead to high transaction cost that discourages many suppliers to enter the market.^18^ Health system and broader contextual factors might affect the scaling up of vaccines such as type of product and disease, health system governance, financing, supply chains, political will, advocacy and trust.^19^ Given the public health benefits that could be attained through increased coverage of new and existing vaccines in LMICs, it is important to understand how the interrelated stages of R&D, launch and scale up of vaccines might be optimised to reduce the overall time from vaccine discovery to uptake.

## What did we observe from the different journeys of COVID-19 and other vaccines?

We compared the end-to-end scale up pathway of 10 COVID-19 vaccines and 8 non-COVID-19 vaccines. We defined the end-to-end pathway into three milestones covering R&D, launch and scale.^20^ R&D refers to the time from vaccine ideation to first approval of the vaccine by a national regulatory authority (in any LMIC). Launch is defined as the time from first approval by a national regulatory authority to first introduction, commercialisation or procurement of the vaccine within any LMIC (outside of a research study). Scale marks the time from first launch within any LMIC to at least 20% uptake of the vaccine across all LMICs.^21^ For the non-COVID-19 vaccines, we referred to Luthra *et al*’s list of Gavi supported vaccines^22^ and purposively selected illustrative vaccines for different types of VPDs and manufactured by different types of developers. For vaccines with different manufacturers, we chose the first manufacturer that received WHO prequalification for better data availability. We have not included an exhausting list of vaccines, which is a limitation and our findings and implications should be interpreted with caution.

Among COVID-19 vaccines the median time taken was 0.90 years (max: 1.68 years) for R&D, 0.05 years (max: 0.31 years) for launch and 0.92 years (max: 0.97 years) for scale. Among non-COVID-19 vaccines the median time taken was 14.16 years (max: 34.02 years) for R&D, 0.62 years (max: 1.33 years) for launch, and 7.53 years for scale (max: 18.01 years). Only two vaccines reached at least 20% target population in LMICs within 1 year after their launch. Noticeably, we also observed that the durations of different milestones varied substantially among non-COVID-19 vaccines.

## What did we learn from COVID-19 vaccines and what lessons can be applied to other vaccines?

### R&D

The time taken for R&D of the COVID-19 vaccines is nearly 16 times shorter than that of the non-COVID-19 vaccines included (table 1). The reduced duration is partly attributed to the application of rapid genome sequencing of the virus and public sharing of information. The SARS-CoV-2 genome was sequenced and publicly shared only 2 weeks after the first cases of COVID-19 were reported to the WHO and potential targets for vaccine development were identified by various labs across the world within the next month.^3^ However, while a common global incentive and public sharing of information can certainly speed vaccine candidate development, this process is largely dependent on the complexity and biology of the infectious agent being studied. In the case of COVID-19, decades of previous research on coronaviruses and genetic vaccine technologies gave investigators a large base of information to help formulate the first vaccine candidates.^23 24^ Efforts to reduce the burden of other infectious diseases could, therefore, benefit from funding to support research and vaccine development platforms. The Coalition for Epidemic Preparedness Innovations (CEPI), for example, recently announced new investment programmes to support the development of novel vaccine platforms for disease ‘X’.^25^ SK Bioscience also announced to invest US$261 million to build the ‘Songdo Global Research & Process Development Center’ by 2025, which aims to cover the entire process from basic research to commercial manufacturing and to ultimately create a global vaccine ecosystem to preemptively respond to new infectious diseases.^26^ Such investments could prove crucial to averting future pandemics.

**Table 1:** R&D, launch and scale timelines by vaccine

Vaccine	R&D (years)#	Launch (years) #	Scale (years)#
COVID-19 vaccines			
Bharat Biotech_COVAXIN	0.52	0.04	NA*
CanSino_Ad5-nCoV	1.06	0.04	NA*
Gamaleya_Sputnik V	0.41	0.14	NA*
Janssen (J&J)_Ad26.COV2.S	0.92	−0.03	0.25
Moderna_Spikevax	0.93	0.01	0.97
Novavax_NVX-CoV2373	1.68	0.00	−0.49
Oxford-AstraZeneca _AZD1222	0.90	0.05	NA*
Pfizer-BioNTech_BNT162	0.90	0.06	0.93
Sinopharm_SARS-CoV-2	0.48	0.06	NA*
Sinovac_Coronavac	0.48	0.31	1.55
Median of COVID-19 vaccines (IQR)	0.90 (0.41)	0.05 (0.03)	0.92 (0.97)
Other vaccines‡			
GSK_Bivalent oral polio	3.99	0.18	6.38
Merck_Ervebo	15.64	−1.47†	NA*
Merck_Gardasil (quadrivalent)	12.67	1.33	11.01
Chengdu Institute of Biological Products_Japanese Encephalitis	34.02	0.50	18.01
Serum Institute India_MenAfriVac	8.42	0.75	0.83
Bio-Pharma_nOPV2	9.38	0.30	NA*
Merck Sharp & Dohme Corp _RotaTeq	23.61	0.73	8.68
Bharat Biotech, CDC, NIH, All India Institute of Medical Sciences, Stanford University, Indian Institute of Science, BMGF_Rotavac	20.23	1.11	0.33
Median of other vaccines (IQR)	14.16 (11.94)	0.62 (0.84)	7.53 (10.43)

1. Milestones: according to the International Development Innovation Alliance’s ‘Insights on Scaling Innovation Framework, we simplified the end-to-end pathway into the following three stages. Two trained researchers collected the date of milestones through reviews of both peer-reviewed and grey literature (sources have been provided in online supplemental appendix 1). Dates were collected in the format day-month-year. For dates with only the year available, we assigned the milestone to be the midpoint of the year (ie, 1 July). For dates with the year and month available, but not the day, we assigned the milestone to be the midpoint of the month (ie, the 15th). If two milestones were reported in same year but without specific date, we assumed the difference to be 0.5 year.^20 21^

1.1 R&D: the time from vaccine ideation to first approval of the vaccine by a national regulatory authority (in any LMIC).

1.2 Launch: the time from first approval by a national regulatory authority to first introduction, commercialisation or procurement of the vaccine within any LMIC (outside of a research study).

1.3 Scale: the time from first launch within any LMIC to at least 20% uptake of the vaccine across all LMICs. LMICs uptake at 20% was selected due to its availability of data for most interventions. For COVID-19 vaccines, the calculation is based on supply (procurement) data (based on the first time the coverage reached 20%); for other vaccines, calculation is based on demand (actual coverage or use) or supply (procurement) data whichever data are available. The denominator is the LMIC population with the VPD (‘target user’ of the vaccine).

*NA denotes data are unavailable or timeline is not yet complete

†Merck_Ervebo vaccine was used as an investigational vaccine under an expanded access programme to help mitigate Ebola outbreak in 2018.

‡Selection of non-COVID-19 vaccines: For the non-COVID-19 vaccines, we referred to Luthra *et al*’s list of Gavi supported vaccines^22^ and purposively selected illustrative vaccines for different types of VPDs and manufactured by different types of developers. For vaccines with different manufacturers, we chose the first manufacturer that received WHO prequalification for better data availability. We have not included an exhausting list of vaccines, which is a limitation and our findings and implications should be interpreted with caution.

LMICs, low-income and middle-income countries; NA, not available; nOPV2, novel oral polio vaccine type 2; R&D, research and development; VPDs, vaccine-preventable diseases.

10.1136/bmjgh-2023-012855.supp1 Supplementary data

Clinical trial and regulatory efficiencies have also contributed to the quick development of COVID-19 vaccines. Several innovative strategies have been used to reduce the time needed for COVID-19 vaccine candidates to demonstrate efficacy and safety and obtain regulatory approval including: (1) rapid identification of clinical trial sites based on the monitoring of COVID-19 infection rates across the world; (2) adaptive clinical trial designs whereby key phases of the trial process are conducted in parallel rather than sequentially; (3) harmonised clinical trial protocols in which similar vaccine candidates from different developers use the same protocol and (4) rolling regulatory reviews that allow developers to submit (and regulators to review) clinical trial data on a continuous rather than intermittent basis.^1 2^ These efficiencies, however, were achieved at a high cost. The US government, for example, spent an estimated US$18–US$23 billion on COVID-19 vaccine development.^27^ Replicating these strategies may, therefore, not be feasible for other infectious diseases not prone to pandemics or for which effective treatments are currently available. Regardless, investments to establish multicentre or regional clinical trial networks where disease burden and clinical trial data are shared in real time could optimise both clinical trial site selection and clinical trial processes, thereby reducing the time and resources needed to obtain a successful vaccine candidate. There might also be additional value in applying data science and artificial intelligence to the mining of clinical trial data and simulations to supplement existing trials.

Partnerships also played an important role in reducing the time needed to develop COVID-19 vaccines. By mid 2021, at least 81 partnerships, targeted at developing a COVID-19 vaccine, had been registered by the WHO about half of which were material transfer partnerships and half knowledge sharing partnerships.^28^ In a material transfer partnership, one partner supplies the vaccine candidate and the other supplies infrastructure to support regulatory approval, manufacturing and distribution whereas in a knowledge sharing partnership both parties work together to develop vaccine candidates, seek regulatory approval and scale up manufacturing and distribution.^29 30^ While the COVID-19 pandemic demonstrated that both types of partnerships can effectively mobilise resources and accelerate vaccine development, knowledge sharing partnerships may be more beneficial to other infectious diseases. These partnerships tend to promote innovations within both the primary and ancillary activities of vaccine development.^28 31 32^ For infectious diseases that receive little funding (eg, neglected tropical diseases), knowledge sharing partnerships may be a useful tool for accelerating innovation with limited resources.

### Launch

The launches of COVID-19 vaccines took about 12 times shorter than that of non-COVID-19 vaccines (table 1). The WHO played an important role in facilitating the rapid launch of COVID-19 vaccines in LMICs. The WHO Emergency Use Listing (EUL) and prequalification programmes have been widely used to determine which vaccine candidates are safe to procure and distribute. WHO conducted expedited, but comprehensive reviews of the safety and efficacy of COVID-19 vaccine candidates to determine their eligibility for EUL or prequalification.^33^ This allowed COVAX, which delivered many vaccines for LMICs during the pandemic, to distribute vaccines to LMICs as quickly as possible. The rapid scale of novel oral polio vaccine type 2 (nOPV2) can also be attributed to the WHO EUL.^17^ More generally, LMICs with limited regulatory capacity and other global health purchasers, such as the Global Fund, refer to WHO prequalification to guide their procurement of vaccines for other infectious diseases.^34^ Given the importance of WHO prequalification/EUL to LMICs during pandemic and non-pandemic times, ongoing efforts should be made to strengthen the prequalification process to increase access to vaccines in LMICs. Such efforts may include improving transparency of the prequalification assessment, developing detailed guidance for stakeholders on navigating the prequalification process, and providing more opportunities for stakeholders to offer input on how the prequalification programme should evolve overtime.^18^

Although the WHO prequalification/EUL programmes helped LMICs receive COVID-19 vaccine doses quickly, the COVID-19 pandemic highlighted the value of LMICs increasing their regulatory capacities. The WHO Global Benchmarking Tool allows national regulatory authorities to assess their level of ‘maturity’ through a comprehensive set of indicators.^35^ The highest maturity levels (levels 3 and 4) denote ‘stable, well-functioning and integrated regulatory systems’ and ‘regulatory systems operating at advanced level of performance and continuous improvement’, respectively.^35^ Coordinated investments to build regional regulatory bodies and strengthen national regulatory bodies could accelerate the approval thereby expediting access to vaccines in future pandemics. Moreover, increased regulatory capacity among LMICs combined with a push for increased manufacturing capacity and voluntary licensing of vaccine products would bring LMICs one step closer to becoming vaccine self-sufficient.^36^ Under such circumstances, LMIC-based manufacturers could produce for their regions under voluntary licensing agreements and individual countries could then approve these vaccines for distribution to their populations. Indeed, as a consequence of the COVID-19 pandemic, numerous vaccine manufacturing capacity initiatives are in development across LMICs. The Partnership for African Vaccine Manufacturing, for example, was created to scale manufacturing of vaccines, therapeutics and diagnostics on the African continent (PAVM website). In addition, the WHO established an mRNA vaccine technology transfer hub in South Africa and corresponding network of LMIC-based technology recipients to build mRNA vaccine production capacity across LMICs.^37^ Such initiatives could help address existing gaps and strengthen vaccine self-sufficiency across LMICs.

Public–private partnerships were crucial to quickly launching COVID-19 vaccines in LMICs.^38^ At the international level, in April 2020, the Access to COVID-19 Tools Accelerator (ACT-A) was formed by a combination of ‘governments, scientists, businesses, civil society, philanthropists and global health organisations’ to support the development and equitable distribution of COVID-19 tests, treatments and vaccines (WHO ACT Accelerator 2023). COVAX—the vaccines pillar of ACT-A led by the WHO, CEPI, Gavi, the vaccine alliance and UNICEF—has distributed more than 1.8 billion COVID-19 vaccine doses across LMICs to date.^39^ Despite the contributions of COVAX, there have been calls for increased inclusion of LMICs in COVAX’s decision-making processes.^40 41^ LMIC governments, for example, have felt insufficiently included in ACT-A’s governance structure leading to inefficiencies in the planning and delivery of COVID-19 tools to these countries.^3^ COVAX as well as public–private partnerships to support access to vaccines for other diseases should include LMICs at the decision-making table so that these countries, which often face the highest burden of infectious diseases, are able to influence how funds and intellectual property are allocated and where research and manufacturing takes place.^42^ Efforts, such as the African Vaccine Acquisition Task Team, can serve as a regional mechanism to ensure access to vaccine.

### Scale

COVID-19 vaccines were over eight times faster to reach 20% uptake across LMICs than that of other vaccines (table 1). While it is hard to argue that COVID-19 has received highest political priority and resources, the pooled procurement mechanisms and mass vaccination campaigns have also contributed to the rapid scale up COVID-19 vaccines. COVAX, for example, pooled the purchasing power of countries worldwide to make at-risk investments in COVID-19 vaccine manufacturing which ultimately resulted in billions of doses being procured and delivered to LMICs. Importantly, however, the full potential of COVAX was undermined by multiple challenges, including lack of consideration and planning for countries to act in self-interest during times of crisis rather than global solidarity. Nonetheless, the COVID-19 pandemic highlighted the value of using pooled procurement mechanisms or other market shaping interventions, which could reduce the transaction cost and risk of manufacturers, to increase timely access to vaccine doses.^43^ Such market shaping mechanisms could be used by LMICs to procure vaccines for other infectious diseases with smaller markets (ie, less demand). With sufficient political will, funding and governance, regional pooled procurement entities could be established among LMIC Ministries of Health to increase access to vaccines and ensure regional vaccine equity.^44^

Despite the efforts in scaling up of COVID-19 vaccines in LMICs, the pandemic revealed inefficiencies in vaccine manufacture, distribution, delivery and demand generation. According to a WHO survey of influenza manufacturers in 2019, the global vaccine manufacture capacity could only vaccinate 25% of the world’s population in the first year.^45^ Additionally, LMICs cited inconsistent vaccine deliveries as a major hindrance to supply chain logistics. More specifically, doses would be delivered inconsistently in massive quantities (close to the expiration date) and without warning. These surges of vaccines would overwhelm available infrastructure (eg, transport vehicles and cold storage) and human resources (eg, trained vaccinators) thus preventing administration of the vaccines before their expiry.^46^ Again, this inefficiency highlights the need to include LMIC stakeholders at the decision-making table so that they may shape global supply chain activities in a manner that is consistent with local resource capacity and preference. Future efforts by global health organisations, donor countries and manufacturers to procure and deliver vaccines for any infectious disease to LMICs should necessarily involve stakeholders from these countries. Other factors that might contribute to reducing wastage include regional manufacturing and capacity building efforts for supply chain management.

Valuable insights regarding the inclusion of LMIC stakeholders at the decision-making table of vaccine procurement and delivery logistics may be drawn from the scale up of nOPV2. nOPV2 received EUL status by the WHO in 2020.^47^ However, by the end of 2019, the Global Polio Eradication Initiative (GPEI) had established a global level nOPV2 working group to ensure LMIC leaders had up to date information on vaccine efficacy and safety data, guidance documents for developing nOPV2 rollout plans, technical documents on administration of the vaccine, and open communication channels with the working group to support country preparations for roll-out.^47^ These efforts by the GPEI to keep all affected countries equally informed and engaged resulted in quick scale up of the vaccine. By the end of 2021, 8 countries had rolled out nOPV2 and 39 more had rolled out plans in development.^47^ Global level working groups similar to that used for the roll-out of nOPV2 should be considered in the future to support efficient and equitable scale up of vaccines for other infectious diseases in LMICs.

Lastly, it is important to note that the rapid development of COVID-19 vaccines has given rise to vaccine hesitancy across the world.^48^ If strategies applied to COVID-19 vaccine development are to be used for the rapid development of other vaccines, then clear and up to date information regarding vaccine science, safety and efficacy must be shared with the public in a timely manner and through trusted outlets to build public trust and promote vaccine uptake.

## Conclusion

Based on the public health impact of infectious diseases, insights gained and lessons learnt from the rapid R&D, launch and scale up of COVID-19 vaccines might have different implications for the vaccines targeting other infectious diseases. Funding the development of novel vaccine platforms, establishing regional clinical trial centres and data sharing networks, and forming public–private knowledge sharing partnerships are all strategies that may accelerate the R&D of new and existing vaccine candidates. In addition, including LMICs in the decision-making processes of global procurement and delivery alliances, strengthening the WHO prequalification and EUL programmes, and investing in LMIC regulatory and manufacturing capacity will likely bring LMICs closer to a state of vaccine self-sufficiency thereby reducing delays to launching new and existing vaccines in these countries. Lastly, creating regional pooled procurement mechanisms between LMICs, including LMICs in global supply chain decision-making, and keeping an open line of clear communication with the public regarding pertinent vaccine information may increase uptake of new and existing vaccines in LMICs and other countries worldwide.

## Data Availability

Data are available in a public, open access repository. All data presented are available from the Launch and Scale Speedometer website: https://launchandscalefaster.org/.
