# Supplementary material for: Comparing research and development, launch, and scale up timelines of 18 vaccines: lessons learnt from COVID-19 and implications for other infectious diseases
Source: BMJ Glob Health. 2023 Sep 11;8(9):e012855. doi: 10.1136/bmjgh-2023-012855 (PMC10496705; doi:10.1136/bmjgh-2023-012855)
Supplement: Supplementary data [file bmjgh-2023-012855supp001.pdf]

Appendix 1

Table S1. Milestone dates, sources, and links for each vaccine included in the analysis

	Ideation			First regulatory approval			First in-country launch			Global uptake of intervention at 20%		
**Vaccine**	**Date**	**Source**	**Link to source**	**Date**	**Source**	**Link to source**	**Date**	**Source**	**Link to source**	**Date**	**Source**	**Link to source**
Bharat Biotech_COVAXIN	2020-06-29	CNBC-TV18. (2020, June 29). India's first COVID-19 vaccine candidate COVAXIN gets DCGI approval; human trials to begin in July	https://www.cnbctv18.com/healthcare/indias-first-covid-19-vaccine-candidate-covaxin-gets-dcgi-approval-6226931.htm	2021-01-03	Press Information Bureau Government of India. (2021, January 3). Press Statement by the Drugs Controller General of India (DCGI) on Restricted Emergency approval of COVID-19 virus vaccine.	https://vaccine.icmr.org.in/images/pdf/HFW-DCGI-authorisation-3rdJanuary.pdf	2021-01-16	Ellis-Petersen, H. (2021, January 16). India begins world's biggest Covid vaccination programme. The Guardian.	https://www.theguardian.com/world/2021/jan/16/india-begins-worlds-biggest-covid-vaccination-programme	#N/A	#N/A	#N/A
CanSino_Ad5-nCoV	2020-01-20	CanSino Biologics. (n.d.). Our Fight Against Covid-19, Timeline.	http://www.cansinotech.com/html/1//156/218/index.html	2021-02-10	Drazen Jorgic. Reuters. 2/10/2021. Mexico approves China's CanSino and Sinovac COVID-19 vaccines.	https://www.reuters.com/article/us-health-coronavirus-mexico-cansino/mexico-approves-chinas-cansino-and-sinovac-covid-19-vaccines-idUSKBN2AA12W	2021-02-26	PTI. (2021, February 28). China rolls out first one-jab Covid-29 vaccine: Report. Economic Times, Healthworld.	https://health.economictimes.indiatimes.com/news/pharma/china-rolls-out-first-one-jab-covid-19-vaccine-report/81257865	#N/A	#N/A	#N/A

Gamaleya_ Sputnik V	2020-06-10	Russian Direct Investment Fund. (2020, June 10). RDIF, Sistema and the Gamaleya National Research Institute of Epidemiology and Microbiology agree on production of Russia's first coronavirus vaccine.	https://rdif.ru/Eng_fullNews/5326/	2020-11-08	NF Gamaleya NITsEM "of the Ministry of Health of Russia	https://grls.rosminzdrav.ru/Grls_View_v2.aspx?routingGuid=6c1f7501-7067-45b3-a56d-95e25db89e97&t	2020-12-29	Litvinova, D. & Calatrava, A. (2020, December 29). Belarus, Argentina start vaccinations with Russian shots. ABC News.	https://abcnews.go.com/Health/wireStory/belarus-starts-covid-19-vaccinations-russian-shots-74945827	#N/A	#N/A	#N/A
Janssen (J&J)_Ad26.COV2.S	2020-03-30	Gorsky, A. (2020, March 30). "I Have Never Seen a Moment So Rich in Collaboration, Ingenuity and Acts of Bravery": Johnson & Johnson Announces It Has Identified a Lead COVID-19 Vaccine Candidate.	https://www.jnj.com/latest-news/johnson-johnson-ceo-alex-gorsky-announces-coronavirus-vaccine-candidate	2021-02-27	Hinton, D. (2021, June 10). Janssen COVID-19 Vaccine EUA Letter of Authorization on 06102021. FDA Memo.	https://www.fda.gov/media/146303/download	2021-02-17	South Africa Government News Agency. (2021, February 18). Everything you need to know about the Johnson & Johnson vaccine. SAnews.gov.za.	https://www.sanews.gov.za/south-africa/everything-you-need-know-about-johnson-johnson-vaccine	2021-05-21	Launch and Scale Speedometer: Vaccine Purchases	https://launchandscalefaster.org/covid-19/vaccinepurchases
Moderna (Spikevax)	2020-01-13	Moderna. (2020, November 16) COVID-19 vaccine	https://www.modernatx.com/sites/default/files/content	2020-12-18	Hinton, D. (FDA). (2021, July 7). Moderna	https://www.fda.gov/media/144636/download	2020-12-20	Reed, M., (2020, December 22). First Moderna	https://www.wcvb.com/article/first-moderna-	2021-12-10	Launch and Scale Speedometer: Vaccine Purchases	https://launchandscalefaster.org/covid-

		(mRNA-1273) Last program update: November 16, 2020.	_documents/mRNA-1273-Update-11-16-20-Final.pdf		COVID-19 Vaccine EUA Letter of Authorization 07072021. FDA.			COVID-19 vaccines given. WCVB Boston.	covid-19-vaccines-given/35042711			19/vaccine purchases
Novavax_NVX-CoV2373	2020-02-26	Novavax.inc press office. (2020, February 26). Novavax Advances Development of Novel COVID-19 Vaccine.	https://ir.novavax.com/2020-02-26-Novavax-Advances-Development-of-Novel-COVID-19-Vaccine	2021-11-01	Australian Government. TGA provisionally approves Novavax (Biocelect Pty Ltd's) COVID-19 vaccine NUVAXOVID	https://www.tga.gov.au/media-release/tga-provisionally-approves-novavax-biocelect-pty-ltds-covid-19-vaccine-nuvaxovid	2021-11-01	Australian Government. TGA provisionally approves Novavax (Biocelect Pty Ltd's) COVID-19 vaccine NUVAXOVID	https://www.tga.gov.au/media-release/tga-provisionally-approves-novavax-biocelect-pty-ltds-covid-19-vaccine-nuvaxovid	2021-05-06	Launch and Scale Speedometer: Vaccine Purchases	https://launchandscalefaster.org/covid-19/vaccinepurchases
Oxford-AstraZeneca_AZD1222_AZ	2020-02-07	The Jenner Institute. (7-Feb-20). Novel Coronavirus vaccine manufacturing contract signed.	https://www.jenner.ac.uk/about/news/novel-coronavirus-vaccine-manufacturing-contract-signed	2020-12-30	Aislinn Laing - Reuters (2020, December 30). Argentine regulator approves AstraZeneca/Oxford COVID-19 vaccine - AstraZeneca. Reuters.	https://www.reuters.com/article/us-health-coronavirus-argentina-astrazen/argentine-regulator-approves-astrazeneca-oxford-covid-19-vaccine-astrazeneca-idUSKBN29421P	2021-01-16	Aleem, M. A. (2021, March 19). Rapid Response: Re: Covid-19: WHO says rollout of AstraZeneca vaccine should continue, as Europe divides over safety. https://doi.org/10.1136/bmj.n728	https://www.bmj.com/content/372/bmj.n728/rr-1	#N/A	#N/A	#N/A
Pfizer-BioNTech_BNT162	2020-01-10	Polack, F. P., Thomas, S. J., Kitchin, N., et al C4591001 Clinical Trial Group	https://www.nejm.org/doi/full/10.1056/NEJMoa2034577	2020-12-02	U.S. Food & Drug Administration (2021, August, 23). FDA Approves	https://www.fda.gov/news-events/press-announcements/fda-	2020-12-24	AP News staff. (2020, December 24). Mexico starts giving first shots of Pfizer-	https://apnews.com/article/mexico-coronavirus-pandemic-coronavirus	2021-11-29	Launch and Scale Speedometer: Vaccine Purchases	https://launchandscalefaster.org/covid-19/vaccinepurchases

		(2020). Safety and Efficacy of the BNT162b2 mRNA Covid-19 Vaccine. The New England journal of medicine, 383(27), 2603–2615. https://doi.org/10.1056/NEJMoa2034577			First COVID-19 Vaccine. FDA News Release.	approves-first-covid-19-vaccine		BioNtech vaccine. AP News.	-vaccine-mexico-city-16105a31023e7ff1cdb7893b452e2906			
Sinopharm_SARS-CoV-2	2020-01-01	Corum, J. & Zimmer, C. (2021, May 7). How the Sinopharm Vaccine Works. The New York Times.	https://www.nytimes.com/interactive/2020/health/sinopharm-covid-19-vaccine.html	2020-06-24	Zhuang, P. (2020, September 25). Coronavirus: WHO backed China's emergency use of experimental vaccines, health official says. South China Morning Post.	https://www.scmp.com/news/china/society/article/3103121/coronavirus-who-backed-chinas-emergency-use-experimental	2020-07-16	Wee, S. & Simões M. (2020, July 16). In Coronoavirus Vaccine Race, China Strays From the Offical Paths. The New York Times.	https://www.nytimes.com/2020/07/16/business/china-vaccine-coronavirus.html	#N/A	#N/A	#N/A
Sinovac_Coronavac	2020-01-01	Sinovac. (2020, April 14). Sinovac Announces Approval of Human Clinical Trial for a Vaccine Candidate	http://www.sinovacbio.com/news/shownews.php?id=1126&lang=en	2020-06-24	Zhuang, P. (2020, September 25). Coronavirus: WHO backed China's emergency use of experiment	https://www.scmp.com/news/china/society/article/3103121/coronavirus-who-backed-chinas-emergency-use-	2020-10-15	Wee, S. (2020, October 16). A Chinese city says it has given some residents a vaccine. The New York Times.	https://www.nytimes.com/2020/10/16/world/a-chinese-city-says-it-has-given-some-residents-a-vaccine.html	2022-05-04	Launch and Scale Speedometer: Vaccine Purchases	https://launchandscalefaster.org/covid-19/vaccinepurchases

		Against COVID-19.			al vaccines, health official says. South China Morning Post.	experimental						
Bivalent Oral Polio Vaccine	2005-10-13	Kew, O. M., Sutter, R. W., de Gourville, E. M., et al. (2005). Vaccine-derived polioviruses and the endgame strategy for global polio eradication. Annual review of microbiology, 59, 587–635. https://doi.org/10.1146/annurev.micro.58.030603.123625	https://pubmed.ncbi.nlm.nih.gov/16153180/	2009-10-09	World Health Organization. Prequalified Vaccines - Vaccine Public Summary Assessment Report.	https://extranet.who.int/pqweb/content/polio-sabin-one-and-three-0	2009-12-15	Harvard Medical School. (September 2018). Cases in Global Health Delivery: Chasing Polio Eradication: Vaccine Development.	https://www.globalhealthdelivery.org/files/ghd/files/ghd-043_polio_vaccine.pdf	2016-05-02	Farrell M, Hampton LM, Shendale S, et al. Monitoring and Validation of the Global Replacement of tOPV with bOPV, April–May 2016, The Journal of Infectious Diseases, Volume 216, Issue suppl_1, 1 July 2017, Pages S193–S201, https://doi.org/10.1093/infdis/jiw558	https://academic.oup.com/jid/article/216/suppl_1/S193/3935039
Ervebo	2004-05-01	Garbutt M, Liebscher R, WahlJensen V, et al. Properties of replication-competent vesicular stomatitis virus vectors	https://www.ncbi.nlm.nih.gov/pmc/articles/PMC400370/	2019-12-19	USFDA. (19 December 19 2019). Approval Letter - ERVEBO.	https://www.fda.gov/media/133757/download	2018-07-01	First FDA-approved vaccine for the prevention of Ebola virus disease, marking a critical milestone in public	https://www.fda.gov/news-events/press-announcements/first-fda-approved-vaccine-prevention-ebola-virus-	#N/A	#N/A	#N/A

		expressing glycoproteins of filoviruses and arenaviruses. J Virol. 2004 May;78(10):5458-65. doi: 10.1128/jvi.78.10.5458-5465.2004.						health preparedness and response	disease-marking-critical-milestone-public-health			
Gardasil	1993-07-01	Caroline McNeil, Who Invented the VLP Cervical Cancer Vaccines?, JNCI: Journal of the National Cancer Institute, Volume 98, Issue 7, 5 April 2006, Page 433	https://academic.oup.com/jnci/article/98/7/433/2522050?login=false	2006-03-01	Gardasil FDA Approval History. Drugs.com	https://www.drugs.com/history/gardasil.html	2007-07-01	PATH	https://path.azureedge.net/media/documents/Global_Vaccine_Intro_Overview_Slides_Final_PATHwebsite_2021AUG17_fx7PZjH.pdf	2018	WHO HPV vaccination coverage	https://immunizationdata.who.int/pages/coverage/hpv.html?CODE=Global&ANTIGEN=&YEAR=
Japanese Encephalitis Vaccine	1954-07-01	Yun SI, Lee YM. Japanese encephalitis: the virus and vaccines. Hum Vaccin Immunother. 2014;10(2):263-79. doi: 10.4161/hv.26902.	https://www.ncbi.nlm.nih.gov/pmc/articles/PMC4185882/	1988-07-01	PATH. (August 2016). Combatting Japanese Encephalitis in Nepal: a public health success story.	https://media.path.org/documents/VAD_je_nepal_case_study_r1.pdf?_gl=1*1hyzum9*_gcl_au*MjE5ODAxNzQyLjE2ODkyMTcwNDc.*_ga*MTE5NTcyMjE0Ny4xNjg5MjE3M	1988-07-01	PATH. (August 2016). Combatting Japanese Encephalitis in Nepal: a public health success story.	https://path.azureedge.net/media/documents/VAD_je_nepal_case_study_r1.pdf	2006-07-01	WHO Global Health Database	https://apps.who.int/immunization_monitoring/globalsummary/timeseries/tscoveragejapenc.html

		Epub 2013 Oct 25.				DQ3*_ga_YBSE7ZKDQM*MTY4OTIxNzA0Ny4xLjAuMTY4OTIxNzA0Ny42MC4wLjA.						
MenAfriVac	2001-07-01	Frasch CE, Preziosi MP, LaForce FM. Development of a group A meningococcal conjugate vaccine, MenAfriVac (TM). Hum Vaccin Immunother. 2012 Jun;8(6):715-24. doi: 10.4161/hv.19619. Epub 2012 Apr 12.	https://pubmed.ncbi.nlm.nih.gov/22495119/	2009-12-01	FDA	https://www.fda.gov/files/vaccines,%20blood%20&%20biologics/published/The-Role-of-CBER-in-the-Success-against-Epidemic-Meningitis-in-Africa---MenAfriVac.pdf	2010-09-01	Compaore, M. (30 November 2010). MenAfrivac, a long-waited vaccine for Burkina Faso.	https://www.who.int/medicines/areas/quality_safety/regulation_legislation/icdra/WD-1_vaccine_BurkinaFaso.pdf?ua=1	2011-07-01	Bwaka, A., Bita, A., Lingani, C., et al. (2019). Status of the Rollout of the Meningococcal Serogroup A Conjugate Vaccine in African Meningitis Belt Countries in 2018. The Journal of infectious diseases, 220(220 Suppl 4), S140–S147. https://doi.org/10.1093/infdis/jiz336	https://www.ncbi.nlm.nih.gov/pmc/articles/PMC6822965/
nOPV2	2011-07-01	KonopkaAnstadt, J. L., Campagnoli, R., Vincent, A., et al (2020). Development of a new oral	https://doi.org/10.1038/s41541-020-0176-7	2020-11-13	Polio Global Eradication Initiative	https://polioeradication.org/wp-content/uploads/2022/06/nOPV2-requirements-overview-	2021-03-01	Duke Global Health Innovation Center. nOPV2 Intervention Research Interviews	https://launchandscalefaster.org/blog/nopv2	#N/A	#N/A	#N/A

		poliovirus vaccine for the eradication end game using codon deoptimization. Npj Vaccines, 5(1). doi:10.1038/s41541-020-0176-7				for-countries-.pdf		(2021). Launch and Scale Speedometer. Duke University				
RotaTeq	1982-07-01	Clark HF, Furukawa T, Bell LM, et al. Immune response of infants and children to low-passage bovine rotavirus (strain WC3). Am J Dis Child. 1986 Apr;140(4):350-6. doi: 10.1001/archpedi.1986.02140180084030.	https://pubmed.ncbi.nlm.nih.gov/3006476/	2006-02-03	U.S. Food and Drug Administration. February 3, 2006 Approval Letter - RotaTeq.	http://wayback.archive-it.org/7993/20170723031528/https://www.fda.gov/BiologicsBloodVaccines/Vaccines/ApprovedProducts/ucm142303.htm	2006-10-27	Goveia M et al. (01 September 2010). RotaTeq: Progress toward Developing World Access. The Journal of Infectious Diseases.	https://academic.oup.com/jid/article/202/Supplement_1/S87/850802	2015-07-01	WHO Global Health Observatory	https://www.who.int/data/gho/data/indicators/indicator-details/GHO/rotavirus-vaccines-completed-dose-(rotac)-immunization-coverage-among-1-year-olds-(-)
Rotavac	1993-11-01	Das BK, Gentsch JR, Hoshino Y, et al. Characterization of the G serotype and genogroup of New Delhi newborn	https://pubmed.ncbi.nlm.nih.gov/8212599/	2014-01-20	Central Drugs Standard Control Organisation	https://cdsco.gov.in/opencms/resources/UploadCDSCOWeb/2018/UploadSmPC/8oral.pdf	2015-03-01	PATH Center for Vaccine Innovation and Access. RotaFlash.	https://www.path.org/programs/center-for-vaccine-innovation-and-access/rotaflash/	2015-07-01	WHO Global Health Observatory	https://www.who.int/data/gho/data/indicators/indicator-details/GHO/rotavirus-vaccines-completed-dose-(rotac)-

		rotavirus strain 116E. Virology. 1993 Nov;197(1): 99-107. doi: 10.1006/viro.1993.1570.										immunization-coverage-among-1-year-olds-(-)
